# New clinical characteristics and novel pathogenic variants of patients with hereditary leukodystrophies

**DOI:** 10.1111/cns.13284

**Published:** 2019-12-29

**Authors:** Juan‐Juan Xie, Wang Ni, Qiao Wei, Huan Ma, Ge Bai, Ying Shen, Zhi‐Ying Wu

**Affiliations:** ^1^ Department of Neurology and Research Center of Neurology in Second Affiliated Hospital Key Laboratory of Medical Neurobiology of Zhejiang Province Zhejiang University School of Medicine Hangzhou China; ^2^ Institute of Neuroscience Zhejiang University School of Medicine Hangzhou China; ^3^ CAS Center for Excellence in Brain Science and Intelligence Technology Shanghai China

**Keywords:** clinical heterogeneity, genetic heterogeneity, hereditary leukodystrophy

## Abstract

**Aim:**

Leukodystrophies are a group of inherited white matter disorders with clinical, genetic, and imaging heterogeneity, which usually pose a diagnostic challenge for physicians. We aimed to identify new clinical characteristics and novel pathogenic variants of hereditary leukodystrophies in this study.

**Methods:**

Whole exome sequencing (WES) was performed in 28 unrelated patients clinically suspected with leukodystrophies. Leukocytes enzyme activity test, electroencephalogram (EEG), electromyography (EMG), and brain MRI were conducted. Functional analysis was performed, and the pathogenicity of variants was classified according to the American College of Medical Genetics and Genomics (ACMG) standards and guidelines.

**Results:**

We made definite diagnosis in 8 probands with 12 pathogenic variants and reported new clinical characteristics and imaging features of these patients. Three novel pathogenic variants were identified, including a microdeletion variant c.2654_2654+3del within *CSF1R*, a nonsense variant c.1321C>T, and a missense variant c.166G>C within *GALC*.

**Conclusion:**

Our results have deepened the understanding of clinical, genetic, and imaging heterogeneity of hereditary leukodystrophies, and expanded the spectrum of pathogenic variants and clinical features.

## 1 INTRODUCTION

Leukodystrophies are a group of inherited white matter disorders with disparate genetic underpinnings that specifically affected the axon‐glia unit.^1^, ^2^ When considering a specific leukodystrophy case, genotype‐phenotype correlation often is not precise.^1^, ^3^ Patients with atypical clinical manifestations were usually misdiagnosed as spastic paraplegia, Charcot‐Marie‐Tooth disease (CMT), multiple sclerosis (MS), or cerebral autosomal dominant with subcortical infarcts and leukoencephalopathy (CADASIL). Therefore, early and accurate diagnosis of leukodystrophies usually poses a challenge to physicians.

Direct sequencing of certain suspected gene associated with leukodystrophies is neither effective nor economic, while whole exome sequencing (WES) can provide convenience to screen candidate genes in a short time.^4^ Discovery of causative genes leads to accurate prognosis and genetic counselling for the patients, and it also helps clinicians to get a better understanding of leukodystrophies.

Here, we performed WES in a cohort of Chinese patients with white matter abnormality of unknown etiology, dementia, or spastic paraparesis without pathogenic variants. We made definite diagnosis in 8 probands carrying 12 pathogenic variants and reported new clinical characteristics and imaging features of them.

## 2 MATERIAL AND METHODS

### 2.1 Patients

Patients who exhibit agnogenic white matter abnormality, unexplained spastic paraparesis, or early‐onset dementia were suggestive of leukodystrophies.^1^ Firstly we screened causative genes for CADASIL, adrenoleukodystrophy, hereditary spastic paraplegia (HSP), and familial Alzheimer's disease (FAD) on these patients in our previous studies,^5^, ^6^, ^7^, ^8^ and patients who carry pathogenic variants were excluded. Then, 28 unrelated patients (probands) were recruited consecutively between March 2015 and August 2018 from the Department of Neurology in Second Affiliated Hospital of Zhejiang University School of Medicine. Among them, 17 individuals had abnormal signals in brain white matter, and the other 11 individuals had been diagnosed with HSP. Leukocytes enzyme activity test, electroencephalogram (EEG), electromyography (EMG), and brain MRI were conducted. Written informed consents were obtained for participants or their legal guardians. The study was approved by the Ethics Committee of the Second Affiliated Hospital.

### 2.2 Whole exome sequencing

Genomic DNAs captured from peripheral blood were sequenced by WES. Details on library preparation, sequencing protocol, bioinformatics analysis, and filtering methods were conducted as described previously.^9^ All filtered variants were further validated by Sanger sequencing on an ABI 3500xL Dx Genetic Analyzer (Applied Biosystems) in the probands and the available family members.

### 2.3 Reverse transcription PCR

RNAs were extracted from leukocyte of proband 2 which carries the *CSF1R* splicing variant and normal controls by RNAiso Plus (Takara). Mutant *CSF1R* mRNA of proband 2, wild type *CSF1R* mRNA (NM_005211) of normal controls, and wild type (WT) *EIF2B3* (NM_020365) mRNA were reversely transcribed into cDNA by PrimeScript™ II 1st Strand cDNA Synthesis Kit (Takara) and amplified by high‐fidelity DNA polymerase KOD‐Plus‐Neo (TOYOBO). EIF2B3 is one of the subunits of eIF2B.^10^ Reduced eIF2B activity enhances the translation of *ATF4* (NM_182810) mRNA due to the presence of upstream open reading frames (uORFs) in its 5′ untranslated regions (UTRs).^11^ In order to evaluate the effects of mutant EIF2B3 on eIF2B, wild type 5′ UTRs of *ATF4* were obtained from a normal control with aforementioned means. Following primers were used: *CSF1R* (5′‐CTCTGAGCAAGACCTGGACAAG‐3′, 5′‐TACTCCCTGTCGTCAACTCC‐3′), *EIF2B3* (5′‐ATGGAATTTCAAGCAGTAGTGATGG‐3′, 5′‐TCAGATCTCCATGAGCT GGTC‐3′) and *ATF4* 5′UTRs (5′‐TTTCTACTTTGCCCGCCCAC‐3′, 5′‐GTTGCGGT GCTTTGCTGGAATC‐3′).

### 2.4 Plasmid constructs

We cloned the WT *EIF2B3* cDNA into p3 × Flag‐cmv‐10 vector (p3 × flag‐EIF2B3) by ClonExpress^®^ II One Step Cloning Kit (Vazyme Biotech). Mutant constructs of *EIF2B3*, c.22A>T, and c.1037T>C were, respectively, introduced into the WT plasmid by site‐directed mutagenesis using KOD‐Plus‐Neo (Toyobo). To verify whether the translation led by *ATF4* mRNA 5′UTRs was affected, we inserted 5′UTR before enhanced green fluorescent protein (EGFP) into the vector pEGFP‐N1 (pEGFP‐ATF4‐5′UTR) like literature described.^11^ All plasmids were fully sequenced after construction or mutagenesis.

### 2.5 Cell culture and transfection

For analysis of eIF2B3 expression, HEK293T cells were cultured at 37°C in Dulbecco's modified Eagle's medium (DMEM) (HyClone) supplemented with 10% fetal bovine serum (GIBCO) and cotransfected with WT or mutant EIF2B3 plasmids with pEGFP‐N1 as exogenous control using Lipofectamine 3000 (Invitrogen) according to the manufacturer's instructions. Simultaneously, since the activity of eIF2B holocomplex could be inhibited by phosphorylated α subunit of eIF2 at Ser51 (eIF2αP),^12^, ^13^ this experiment was also used to detect eIF2α phosphorylation level to evaluate the effects of mutant EIF2B3 on eIF2B indirectly. For study on the ATF4‐5′UTR‐linked report, HEK293T cells were cotransfected with pEGFP‐ATF4‐5′UTR and p3 × flag‐EIF2B3.

### 2.6 Western blot

HEK293T cells were harvested for 48 hours after transfection, then collected and lysed. Protein samples were separated by 10% SDS‐PAGE and transferred to a PVDF membrane (Biorad). The membrane was incubated with mouse anti‐Flag (Abmart), mouse anti‐GFP (Santa Cruz biotechnology), rabbit anti‐eIF2α (Cell signaling technology), rabbit anti‐eIF2αP (Cell signaling technology), and mouse anti‐actin (Santa Cruz biotechnology) followed by horseradish peroxidase (HRP)‐conjugated secondary antibody (Merck Millipore). Then, the protein was visualized by enhanced chemiluminescent substrates (Thermo Scientific).

## 3 RESULTS

### 3.1 Genetic findings and pathogenicity classification of variants

**Figure 1:**
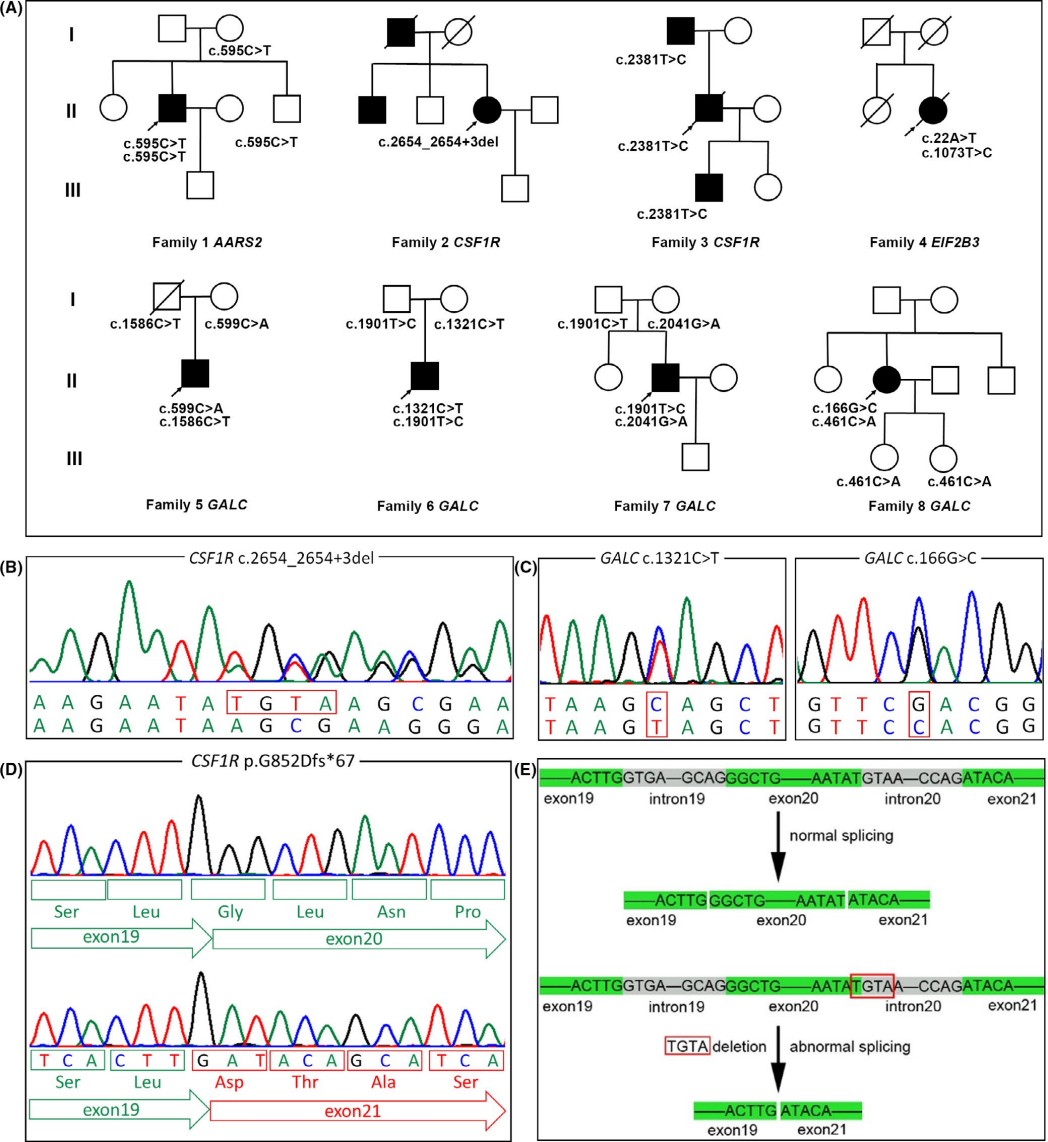
Pedigrees and novel mutations. A, Pedigree charts of the eight families. Squares indicate males, circles indicate females, black symbols indicate affected individuals, and arrows indicate the probands. B and C, Sequencing chromatograms of three novel variants. D and E, The sequencing chromatogram of c.2654_2653+3del within *CSF1R* and schematic diagram of splicing results

After variant screening via WES and verification by Sanger sequencing, we found 12 distinct variants (Table 1) in eight unrelated patients (Figure 1A). Among these 12 variants, three variants (Figure 1B,C) are novel and absent in dbSNP, gnomAD, and ExAC. All of 12 variants are absent in our WES database that contain 500 Chinese controls. As shown in Table 1, c.2654_2654+3del within *CSF1R* is a microdeletion, c.1321C>T within *GALC* is a nonsense variant, and c.166G>C in *GALC* is a missense variant predicted to be deleterious by SIFT, Polyphen‐2, and CADD. According to ACMG, the variant c.166G>C within *GALC* is a variant of uncertain significance. However, the GALC enzyme activity of leukocytes in proband 8 carrying this variant decreased to 2.77 nmol/17 h mg (normal range 12.89‐100.93 nmol/17 h mg protein), and thus, we inferred that this variant is pathogenic. The microdeletion variant c.2654_2654+3del located in the splicer donor site of exon 20 in *CSF1R*, which was confirmed to affect mRNA processing. The RT‐PCR fragment spanning exons 18‐22 of *CSF1R* across the microdeletion yielded two products including the WT fragment (349 bp) and the smaller one (near 250 bp). The smaller one was found to contain a 100 bp in‐frame deletion corresponding to the entire length of exon 20, presenting as the total skipping of exon 20. The junction between nucleotides c.2554 (exon 19) and c.2655 (exon 21) gave rise to an acid substitution (glycine to aspartic) at residue 852 followed by a frameshift that was predicted to lead to the premature termination of translation (p.G852Dfs*67) (Figure 1D,E).

**Table 1:** Twelve pathogenic variants identified in 8 probands with hereditary leukodystrophies

Proband No.	Gene	Nucleotide change	Amino acid change	Genotype	gnomAD	ExAC	SIFT	Polyphen‐2	CADD	ACMG
1	*AARS2*	c.595C>T	p.R199C	Hom	8.9e‐05	0.0001	D	Pro_D	D	P(PS1+PM1+PM2+PM3+PP1+PP3+PP5)
2	*CSF1R*	**c.2654_2654+3del**	p.G852Dfs*67	Het	0	0	NA	NA	NA	P(PVS1+PM1+PM2+PM4+PP3)
3	*CSF1R*	c.2381T>C	p.I794T	Het	4.1e‐06	8.2e‐06	D	Pro_D	D	P(PS3+PM1+PM2+PP3+PP4+PP5)
4	*EIF2B3*	c.22A>T	p.M8L	Het	0.0001	9.8e‐05	T	B	D	US(PM2+PM3)
4	*EIF2B3*	c.1037T>C	p.I346T	Het	0	0	T	B	D	LP(PM1+PM2+PP3+PP5)
5	*GALC*	c.599C>A	p.S200X	Het	0	0	NA	NA	D	P(PVS1+PM2+PP1+PP3+PP5)
5	*GALC*	c.1586C>T	p.T529M	Het	9.8e‐05	6.6e‐05	T	Pro_D	D	LP(PM1+PM2+PM3+PP1+PP3+PP5)
6	*GALC*	**c.1321C>T**	**p.Q441X**	Het	0	0	NA	NA	D	LP (PM1+PM2+PM3+PM4+PP3)
6&7	*GALC*	c.1901T>C	p.L634S	Het	0.0006	0.0007	D	Pro_D	D	LP(PM2+PM3+PP1+PP3+PP5)
7	*GALC*	c.2041G>A	p.V681M	Het	0.0002	0.0002	D	Pro_D	D	LP(PM2+PM3+PP1+PP3+PP5)
8	*GALC*	**c.166G>C**	**p.D56H**	Het	0	0	D	D	D	P (PS3+PM1+PM2+PM3+PP3)
8	*GALC*	c.461C>A	p.P154H	Het	1.219e‐05	8.3e‐06	D	Pro_D	D	LP(PM1+PM2+PM3+PP1+PP3)

Note

Novel variants are in bold.

Abbreviations: ACMG, American College of Medical Genetics and Genomics; B, benign; D, damaging, deleterious, or disease‐causing; ExAC, Exome Aggregation Consortium; gnomAD, The Genome Aggregation Database; LP, likely pathogenic; NA, not applicable; P, pathogenic; Polyphen‐2, Polymorphism Phenotyping v2; Pro_D, probably damaging; SIFT, Sorting Tolerant From Intolerant; T, tolerated; US, uncertain significance.

**Figure 2:**
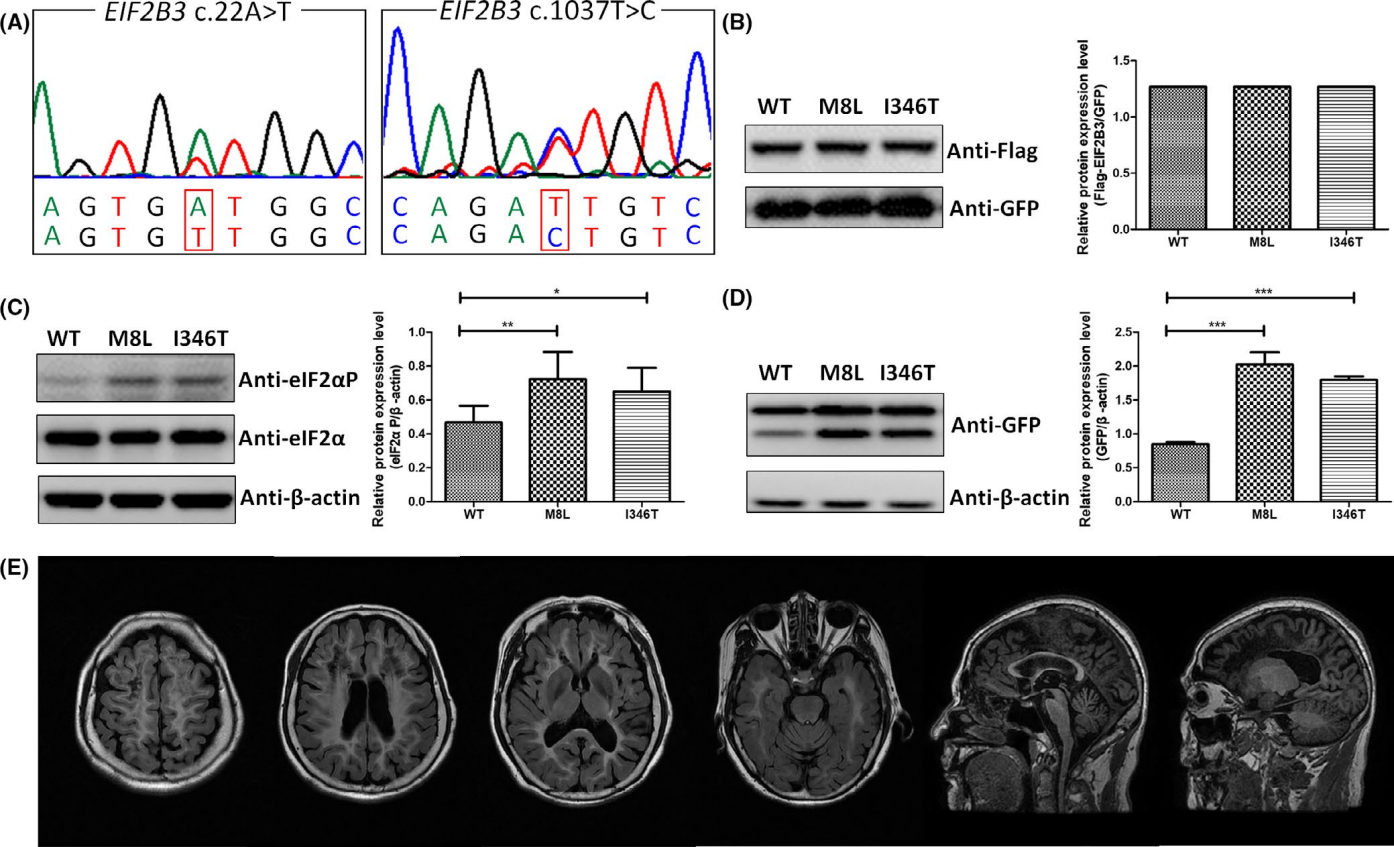
Functional analysis of variants c.22A>T and c.1037T>C within *EIF2B3* and brain MRI of the patient. A, Sequencing chromatograms of variants c.22A>T (p.M8L) and c.1037T>C (p.I346T) within *EIF2B3*. B, HEK293T cells were cotransfected with p3 × flag‐EIF2B3 (WT or mutant) with pEGFP‐N1 as exogenous control. Neither p.M8L nor p.I346T destabilized the protein appreciably. C, Both p.M8L and p.I346T enhanced eIF2α phosphorylation without increase of total eIF2α. *P < .05 and **P < .01. D, HEK293T cells were cotransfected with p3 × flag‐EIF2B3 (WT, or mutant) with (pEGFP‐ATF4‐5′UTR). After incubated with anti‐GFP antibody, the upper bands reflected the fusion protein containing the product translated from the AUG within *ATF4* 5′UTR and EGFP, and the lower bands reflected only EGFP, which were regulated by the uORFs in *ATF4* 5′UTR. Both p.M8L and p.I346T increased the level of expression of EGFP than WT. ***P < .001. E, Brain MRI of proband 4 at her 60 showed extensive white matter hyperintense on FLAIR and markedly hypointense on T1‐weighted images

In the remaining nine known variants, eight variants (Figure 2A right, Figure S1) were previously reported as pathogenic variants, except *EIF2B3* c.22A>T (Figure 2A, left). Given that *EIF2B3* c.1037T>C (Figure 2A, right) was a known pathogenic variant, we investigated whether the functional influence of c.22A>T was similar to that of c.1037T>C. From our study, neither c.22A>T nor c.1037T>C was identified to appreciably destabilize the protein (Figure 2B). Both c.22A>T and c.1037T>C enhanced eIF2α phosphorylation without increase of total eIF2α (Figure 2C) and increased the level of expression of EGFP regulated by the uORFs (Figure 2D). In addition, this patient had the onset of cognitive deterioration and ataxia at 57, with a bilateral, diffuse and symmetric involvement of the cerebral white matter (Figure 2E). She was bedridden with two fractures because of her unsteady walk. Her disease lasted 4 years, and she died at 61. Combined the results of functional experiment with diagnostic clinical manifestations, we deduced that *EIF2B3* c.22A>T was also a disease‐causing variant.

### 3.2 Novel clinical and imaging features

Ultimately, 8 probands were diagnosed as hereditary leukodystrophies, including *AARS2*‐related adult‐onset leukoencephalopathy with axonal spheroids and pigmented glia (ALSP) (proband 1), *CSF1R*‐related ALSP (probands 2 and 3), leukoencephalopathy with vanishing white matter (VWM) (proband 4), and Globoid cell leukodystrophy (GLD) (probands 5‐8). Their primary clinical manifestations were summarized in Table 2. Novel clinical and imaging features are described as follows.

**Table 2:** Clinical and imaging features of 8 probands identified with hereditary leukodystrophies

Proband No.	Sex	Age at onset	Age at present	Gene	Variants	Predominant symptoms	MRI appearances	Diagnosis
1	M	NA	43	*AARS2*	c.595C>T; c.595C>T	Learning difficulty, declining in memorizing and calculating, ataxia, nystagmus, and knee aching	Parietal lobes atrophy, WM abnormality in posterior horns of the LV, posterior CC, and splenium	*AARS2*‐related ALSP
2	F	43	50	*CSF1R*	**c.2654_2654+3del**	Memory and personality change, inattention and acalculia, gait difficulties, dysarthria, and dysphagia	Frontal prominence WM change, thin CC, cortical atrophy	*CSF1R*‐related ALSP
3	M	37	38	*CSF1R*	c.2381T>C	Memory and cognitive regression, gait difficulties, personality change	Widespread T2 hyperintensity in WM and cortical atrophy	*CSF1R*‐related ALSP
4	F	57	61 dead	*EIF2B3*	c.22A>T; c.1037T>C	Dizziness, dementia, gait abnormality, ataxia, and positive Babinski signs	Extensive WM T2 hyperintensity and T1 hypointensity, thin CC, and cerebellar atrophy	VWM
5	M	1	23	*GALC*	c.599C>A; c.1586C>T	Febrile seizures, unstable walking, cognitive regression, and visual field defect	Partly confluent T2 hyperintensity in WM near LV horns and posterior limbs of internal capsule	GLD
6	M	12	28	*GALC*	**c.1321C>T;** c.1901T>C	Shuffling gait, spastic paraplegia, and ataxia	No visible abnormality	GLD
7	M	26	31	*GALC*	c.1901T>C; c.2041G>A	Spastic paraplegia	No visible abnormality	GLD
8	F	35	46	*GALC*	**c.166G>C;** c.461C>A	Shuffling gait and asymmetric weakness in lower limbs	Symmetric corticospinal tract T2 hyperintensity	GLD

Note

Novel variants are in bold.

Abbreviations: ALSP, adult‐onset leukoencephalopathy with axonal spheroids and pigmented glia; CC, corpus callosum; GLD, globoid cell leukodystrophy; LL, lateral ventricles; VWM, leukoencephalopathy with vanishing white matter; WM, white matter.

**Figure 3:**
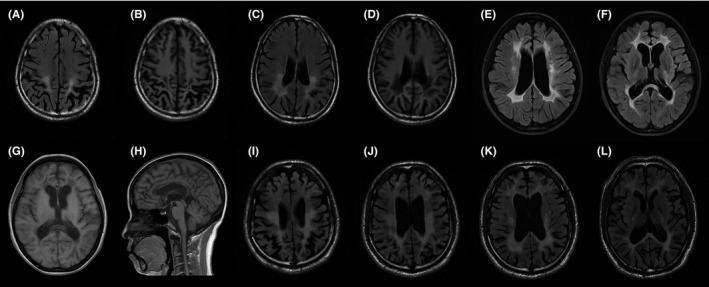
Brain MRI of patients with ALSP. A, B, C and D, MRI of proband 1 at his 43 showed the broadening and deepening of sulus with occipital prominence, and occipital periventricular white matter hyperintense on FLAIR and hypointense on T1‐weighted images. E, F, G and H, MRI of proband 2 at her 46 showed the white matter adjacent to the lateral ventricle hyperintense on FLAIR and hypointense on T1‐weighted images, and thinning of the corpus callosum. I, J, K and L, MRI of proband 3 at his 38 showed widespread white matter hyperintense and obvious cortical atrophy on FLAIR

The *AARS2*‐related ALSP patient (proband 1) was a 43‐year‐old farmer with pain in the left knee for 5 years and numbness in the right lower limb for 1 year, but still able to do his daily farm work. There was no discernible change of cognition from his wife's point of view though he had a history of learning difficulty in childhood. He was diagnosed with left anterior cruciate ligament injury, medial collateral ligament injury, and lumbar disk herniation. Brain MRI examination at age 43 indicated cortical atrophy, most pronounced in bilateral parietal lobes (Figure 3A,B). Fluid‐attenuated inversion recovery (FLAIR) hyperintensity and T1‐wighted images hyperintensity predominantly occur near the posteior horns of lateral ventricle (Figure 3A‐D).

The brother of a *CSF1R*‐related ALSP patient (proband 2) had similar, but much milder, symptoms. The other *CSF1R*‐related ALSP patient (proband 3) received allogeneic stem cell transplantation and died of graft‐versus‐host disease and uncontrolled infection 15 days after the transplantation. His father carried the same pathogenic variant but was not unresponsive and sluggish until he was 60 years old, 23 years later than proband 3. The MRI of the proband 2 at her 46 showed confluent demyelination changes of the white matter adjacent to the lateral ventricles symmetric cortical atrophy (Figure 3E‐G) and thinning of the corpus callosum (Figure 3H), which was less severe than the MRI of proband 3 (Figure 3I‐L).

**Figure 4:**
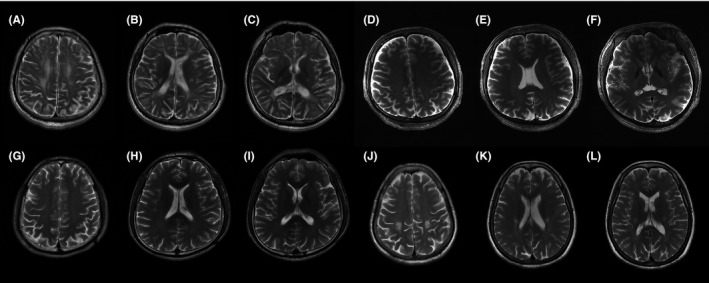
T2‐weighted MRI on the same three levels (centrum semiovale, corona radiate, and internal capsule) of patients with GLD. A, B and C, MRI of proband 5 at his 21 showed partly confluent hyperintense areas in white matter near the lateral ventricle and posterior limbs of internal capsule. D, E and F, MRI of proband 6 at his 28 showed no visible abnormality. G, H and I, MRI of proband 7 at his 28 showed no visible abnormality. J, K and L, MRI of proband 8 at her 46 showed symmetric hyperintense in bilateral corticospinal tracts

Juvenile (proband 6) and adult‐onset (probands 7 and 8) GLD patients were all diagnosed with spastic paraplegia before. EMG results of proband 6 and 7 did not show obvious peripheral nerve injury while proband 8 showed dysmyelination of peripheral nervous system, which led to his diagnosis of CMT. From probands 5 to 8, the GALC enzyme activities in leukocytes were 3.41 nmol/17 h mg, 2.07 nmol/17 h mg, 5.9 nmol/17 h mg, and 2.77 nmol/17h mg (normal range 12.89‐100.93 nmol/17 h mg), respectively. T2‐weighted MRI of probands 5‐8 on the level of centrum semiovale, corona radiate, and internal capsule were presented in Figure 4. There was no visible abnormality in the brain MRI images of proband 6 (Figure 4D‐F) and 7 (Figure 4G‐I) when they were 28 years old. Two consecutive brain MRI of proband 8 were performed at the ages of 41 and 46, respectively, showing no significant progression. The image at the age of 46 showed only involvements of symmetric corticospinal tract (Figure 4J‐L).

## 4 DISCUSSION

It is well established that hereditary leukodystrophies are highly heterogeneous disorders. In this study, we made definitive genetic diagnosis for 8 probands with rare hereditary leukodystrophies by WES and identified 12 pathogenic variants including three novel ones. The patients we described here did show a significant variability of phenotypes.

*AARS2* and *CSF1R* are two uncovered genes responsible for ALSP. Most patients diagnosed with ALSP shared cognitive decline, neuropsychiatric disturbance, and pyramidal and extrapyramidal signs. *AARS2*‐related ALSP is an autosomal recessive neurodegenerative disease while *CSF1R*‐related ALSP is inherited in a dominant manner. Clinical features of ALSP are very heterogeneous, showing a significant intra‐ and inter‐kindred variability of phenotype and disease duration.^14^ We reported one *AARS2*‐related ALSP and two *CSF1R*‐related ALSP cases. The *AARS2*‐related ALSP patient shared the same *AARS2* pathogenic variant with a Turkey middle‐aged patient who presented a rapid progression and died in 1 year after symptoms onset.^15^ But there were no evident dystonia, dysarthria, cognitive deterioration, or neuropsychiatric symptoms in our patient as the Turkey patient presented.^15^ The two *CSF1R*‐related ALSP patients showed great clinical heterogeneity, especially in the patients with *CSF1R* c.2381T>C. The proband (son) had disease onset 23 years earlier than his father. These phenomena reminded us that even patients carrying same pathogenic variants would present totally different phenotypes. The remarkable distinction of clinical manifestation among these genotype‐identical ALSP patients may be due to environmental or other genetic factors that play an important role and cannot be replaced.

Leukoencephalopathy with VWM is an autosomal recessive neurodegenerative disease due to pathogenic variants in the five genes (*EIF2B1* to *5*) encoding the five subunits of the eucaryotic initiation factor 2B (eIF2B).^16^ The variation in disease severity is extremely wide with stress‐provoked episodes of rapid deterioration.^1^ Survival increased with increasing age of onset and 80% of patients with an age of onset over 5 years were expected to be without severe disability at 14 years of disease evolution.^16^ In this study, we identified c.22A>T and c.1037T>C variants within *EIF2B3* in the proband of family 4 and firstly confirmed the pathogenicity of c.22A>T, which was never reported to be associated with VWM. Both of c.22A>T and c.1037T>C were indirectly proved to inhibit eIF2B activity.^11^, ^12^, ^13^ However, the proband had the onset symptom at 57 and died at 61. Such a short duration was inconsistent with the literature. The two fractures could act as factors provoking the episodes of rapid neurological deterioration.

Globoid cell leukodystrophy comprises a spectrum with more severe infantile‐onset disease to milder juvenile or adult‐onset disease. Juvenile and adult‐onset GLD patients were easily misdiagnosed as spastic paraplegia. Globoid cell leukodystrophy is the most common leukodystrophy in our study, and all four GLD patients presented pyramidal signs. Proband 6 and 7 shared one same pathogenic variant, c.1901T>C in *GALC*, which was supposed to contribute to a mild phenotype.^17^ However, the phenotype of proband 6 was more severe than that of proband 7, and we supposed that the nonsense variant c.1321C>T was more deleterious than the missense variant c.2041G>A. It should be noted that there was no significant abnormality in brain MRI of proband 6 and 7 at the age of 28, while another 55‐year‐old asymptomatic individual carrying homozygous c.1901T>C showed selective pyramidal tract impairment in brain MRI.^18^ These MRI features suggested that the demyelinating degree in conventional MRI may not reflect the severity of clinical symptoms in GLD patients.

In summary, we made definite diagnosis in 8 probands with 12 pathogenic variants including three novel ones and reported new clinical characteristics and imaging features of these patients. Our results have deepened the understanding of clinical, genetic, and imaging heterogeneity of hereditary leukodystrophies and expanded the spectrum of pathogenic variants and clinical profiles.

## CONFLICT OF INTEREST

All authors reported no potential conflicts of interest.
