# Supplementary figures and images for: New clinical characteristics and novel pathogenic variants of patients with hereditary leukodystrophies
Source: CNS Neurosci Ther. 2019 Dec 29;26(5):567–75. doi: 10.1111/cns.13284 (PMC7163788; doi:10.1111/cns.13284)

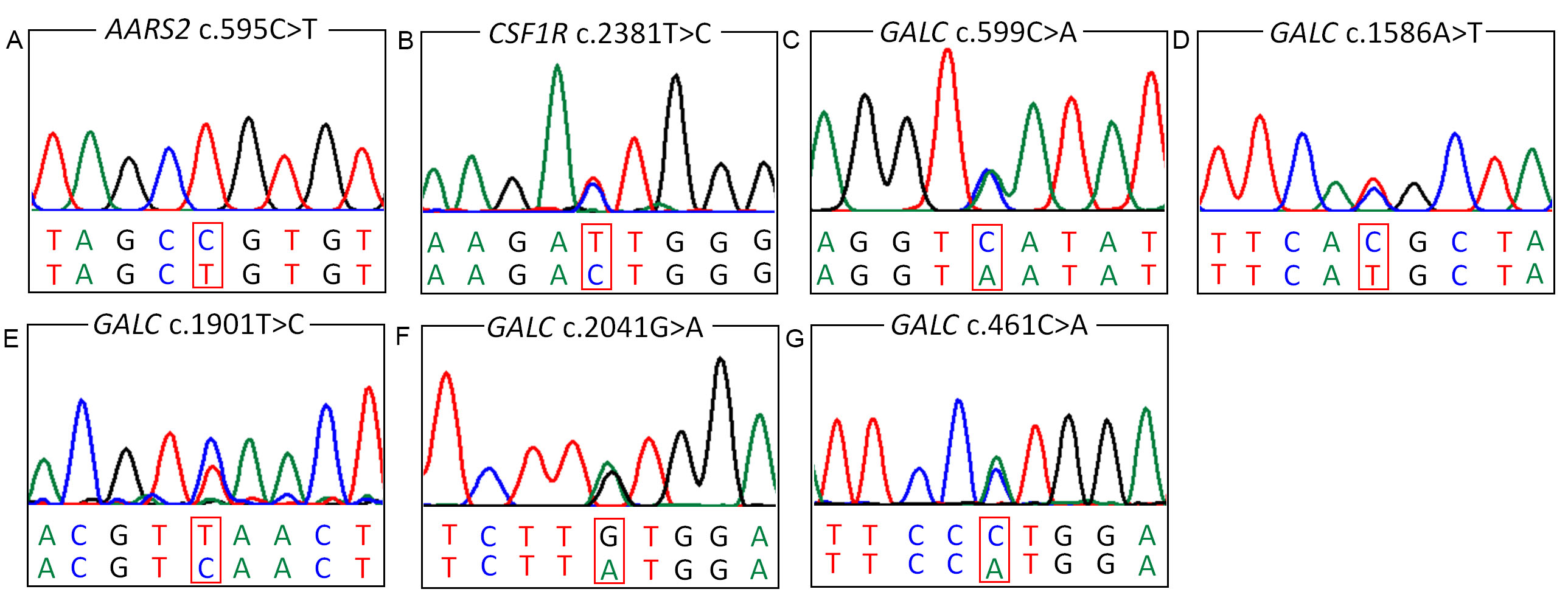


**Supplementary figure 1**. Seven known variants identified in this study.

Supplement: Supplementary file 1 [file CNS-26-567-s001.docx]
